# 4-Methylumbeliferone Treatment at a Dose of 1.2 g/kg/Day Is Safe for Long-Term Usage in Rats

**DOI:** 10.3390/ijms24043799

**Published:** 2023-02-14

**Authors:** Kateřina Štěpánková, Dana Mareková, Kristýna Kubášová, Radek Sedláček, Karolína Turnovcová, Irena Vacková, Šárka Kubinová, Pavol Makovický, Michaela Petrovičová, Jessica C. F. Kwok, Pavla Jendelová, Lucia Machová Urdzíková

**Affiliations:** 1 Institute of Experimental Medicine, Czech Academy of Sciences, 14220 Prague, Czech Republic; 2 Department of Neuroscience, Second Faculty of Medicine, Charles University, 15006 Prague, Czech Republic; 3 Department of Mechanics, Biomechanics and Mechatronics, Faculty of Mechanical Engineering, Czech Technical University in Prague, 16000 Prague, Czech Republic; 4 Institute of Physiology, Czech Academy of Sciences, 14220 Prague, Czech Republic; 5 Institute of Physics, Czech Academy of Sciences, 18221 Prague, Czech Republic; 6 Department of Biology, Faculty of Education, J. Seyle University, SK-94501 Komarno, Slovakia; 7 School of Biomedical Sciences, Faculty of Biological Sciences, University of Leeds, Leeds LS2 9JT, UK

**Keywords:** hyaluronan, 4-methylumbelliferone, chondroitin sulphates, neuroplasticity

## Abstract

4-methylumbelliferone (4MU) has been suggested as a potential therapeutic agent for a wide range of neurological diseases. The current study aimed to evaluate the physiological changes and potential side effects after 10 weeks of 4MU treatment at a dose of 1.2 g/kg/day in healthy rats, and after 2 months of a wash-out period. Our findings revealed downregulation of hyaluronan (HA) and chondroitin sulphate proteoglycans throughout the body, significantly increased bile acids in blood samples in weeks 4 and 7 of the 4MU treatment, as well as increased blood sugars and proteins a few weeks after 4MU administration, and significantly increased interleukins IL10, IL12p70 and IFN gamma after 10 weeks of 4MU treatment. These effects, however, were reversed and no significant difference was observed between control treated and 4MU-treated animals after a 9-week wash-out period.

## 1. Introduction

4-methylumbeliferone (4MU) is used in several European countries under the name ‘hymecromone’ as a treatment for biliary disorders, based on its choleretic and biliary anti-spasmodic activity. The hepatocellular formation of bile is dependent on the active secretion of bile salts (i.e., bile-dependent pathway) and the active transport of sodium ions across the canalicular membrane (bile-independent pathway), which represents a luminal meshwork of tubules between adjacent hepatocytes [1]. This site is important for primary bile formation [2]. It has been suggested that the choleretic activity of 4MU is mediated by the active transport of sodium ions into the bile capillaries and thus enhances the bile flow, which is not dependent on the osmotic force of bile acids [3]. 4MU rapidly decreases the concentration of bicarbonate and chloride ions in bile and thus increases the cation anion gap in the bile duct. The mechanism is believed to be dependent on the conversion of 4MU to the 4-methylumbelliferyl glucuronide in the liver. 4-methylumbelliferyl glucuronide presented in its anionic form is then coupled with sodium ions and actively secreted to the biliary tree, and water passively follows [3]. In addition to its choleretic activity, 4MU is also an inhibitor of hyaluronan (HA) synthesis [4], and HA has been shown to be a potential cure for a number of diseases through the inhibition of HA synthesis, ranging from reducing cancer metastasis in oncologic conditions through ischaemia reperfusion injury to non-alcoholic steatohepatitis. An overview of 4MU doses, 4MU administration time and experimentally treated diseases is summarised in the Supplementary Materials (Table S1).

Recently, it has been proposed that 4MU may serve as a novel plasticity treatment for central nervous system conditions through perineuronal net (PNN) modulation [5,6,7,8]. PNNs are extracellular matrix (ECM) structures that primarily surround brain inhibitory parvalbumin interneurons and spinal motoneurons. These structures stabilise the established neuronal connections and thus limit the plasticity in the matured CNS [9] by restricting plastic changes from environmental adaptation [10]. HA and chondroitin sulphates (CSs) are key components of the PNNs and they share one common monosaccharide residue, glucuronic acid. The binding of 4MU to glucuronic acid reduces the production of UDP-glucuronic acid for HA synthesis [11]. We have previously shown that oral treatment of 4MU reduces PNNs and thus increases the neuroplasticity after spinal cord injury [6] and enhances memory [5]. Besides the experimental application, there are several clinical trials using 4MU. The treated conditions, doses and the current status of the trials are given in the Supplementary Materials (Table S2).

Due to its property of reducing perineuronal nets, 4MU appears to be a drug with potential for many CNS pathologies such as CNS trauma, memory disorders and neurodegenerative diseases [5,6,12,13]. The pharmacokinetics of 4MU in human and rats have been reported when trials were performed for cholestasis [14,15]. However, long-term administration of 4MU at 1.2 g/kg/day, such as that used in spinal cord injury treatment, could potentially develop adverse side effects. It is therefore important to access the effect of long-term 4MU treatment in other organs.

The objectives of this pharmacology study were to identify the possible adverse pharmacological properties of 4-MU that could be relevant for future long-term application in spinal cord injury models. 

The current study characterises the effects mediated by the long-term application (10 weeks) of orally administered 4MU at a dose of 1.2 g/kg/day on CNS, liver, spleen, heart, small intestine, bone marrow and blood. We also studied the biomechanical changes in tendons and skin due to the prevalence of HA and CSPGs in these tissues. Finally, the effect of a 2-month long wash-out period was also examined. The results demonstrate that long-term 4MU administration induces minimal adverse effects in organs and does not lead to any irreversible effects. During the 10-week treatment, we observed decreased levels of HA and CSPGs, significantly increased bile acids corresponding with the 4MU-mediated choleretic activity, as well as increased blood sugars and proteins a few weeks after 4MU administration. Significantly increased interleukins IL10, IL12p70 and IFN gamma after 10 weeks of 4MU treatment were also observed. However, no significant differences were found between control-treated and 4MU-treated animals after the 9-week wash-out period. 

## 2. Results

### 2.1. 1.2 g/kg/Day Dose of 4MU Downregulates HA throughout the Body

**Figure 1:**
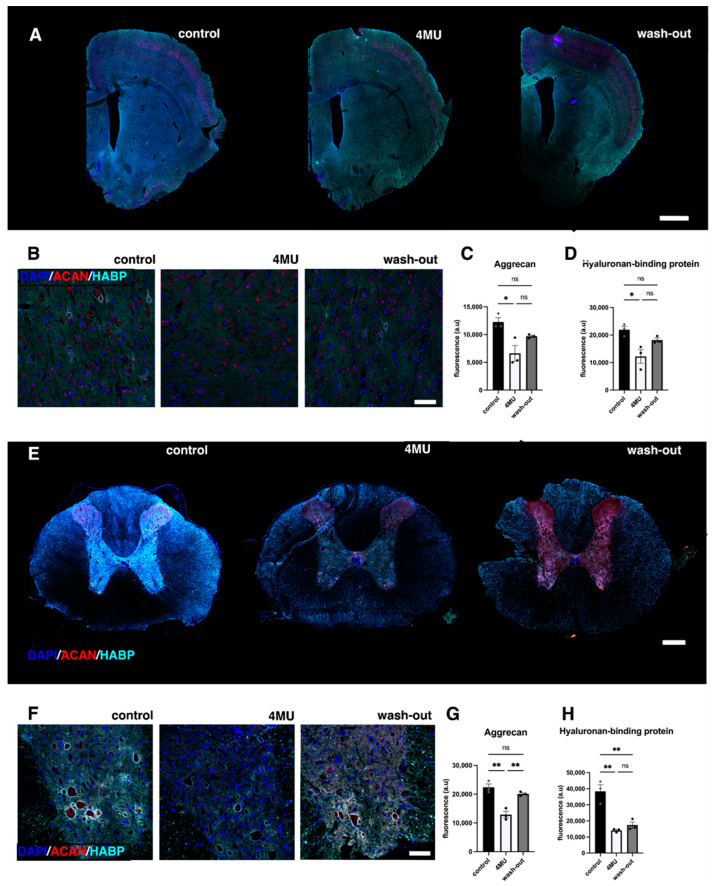
4MU administered at a dose of 1.2 g/kg/day downregulates HA as well as PNNs in brain and spinal cord after 10 weeks of administration. (**A**) Representative confocal images showing HA- and ACAN-positive areas in brain sections in placebo-fed animals, 4MU-treated animals and animals after 9 weeks of wash-out period. Scale bar: 1000 µm. (**B**) Representative confocal images showing detail of the brain cortex. In 4MU-treated animals, HA and ACAN positivity is reduced compared to untreated controls and partially returns after the 9 week wash-out period. Scale bar: 50 µm. (**C**,**D**) Quantification of ACAN (red) and HABP signal in the brain cortex. The values are the means ± SEM for *n* = 3 in each group. (**E**) Representative confocal images showing HA (turquoise)-, ACAN (red)- and DAPI (blue)-positive areas in spinal cord sections in placebo-fed animals, 4MU-treated animals and animals after 9 weeks of wash-out. Scale bar: 200 µm. (**F**) Representative confocal images showing detail of the spinal ventral horns. In 4MU-treated animals, HA and ACAN positivity is reduced compared to untreated controls and partially returns after 9 weeks of wash-out, as observed in the brain. Scale bar: 50 µm. (**G**,**H**) Quantification of ACAN- and HABP-positive signals in spinal grey matter. The values are the means ± SEM; * *p* < 0.05, ** *p* < 0.01 by one-way ANOVA with Tukey’s multiple comparisons test, *n* = 3 in each group. ns: no significance.

**Figure 2:**
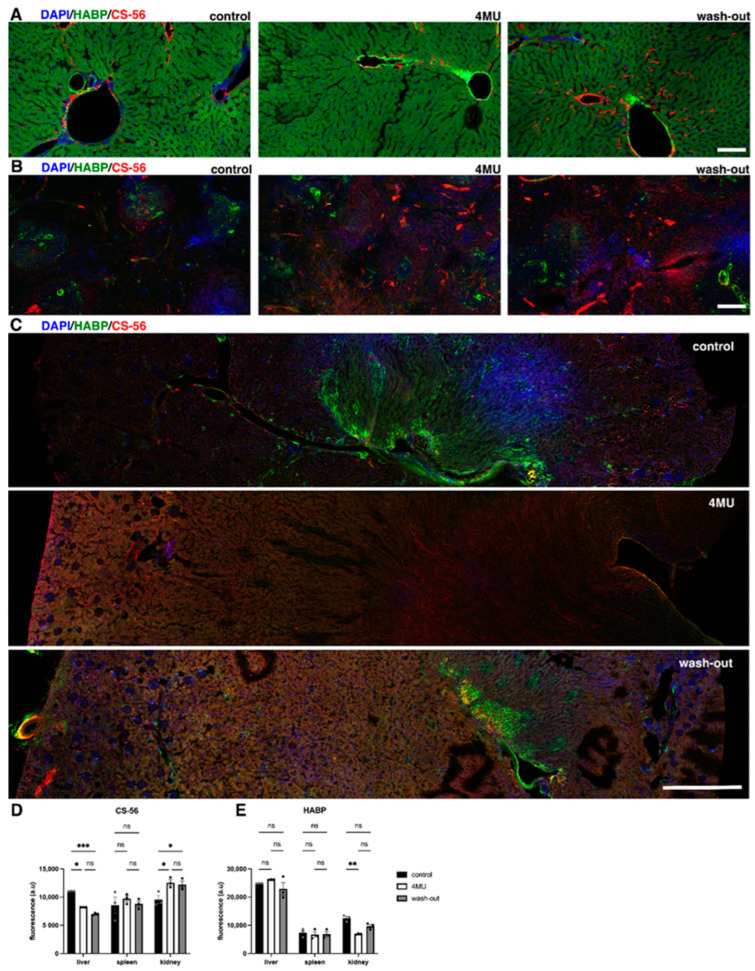
4MU administered at a dose of 1.2 g/kg/day leads to downregulation of HA and CSPGs throughout the body. (**A**) Representative confocal images showing HA- and CSPG-positive areas in (**A**) liver, scale bar 100 µm, (**B**) spleen, scale bar 200 µm, and (**C**) kidney, scale bar 1000 µm, in placebo-fed animals, 4MU-treated animals and animals after 9 weeks of wash-out, indicating downregulation of both markers and the return of signal after 9 weeks of wash-out. (**D**,**E**) Quantification of CS-56- and HABP-positive signals in the kidney, spleen and liver sections. The values are the means ± SEM, * *p* < 0.05, ** *p* < 0.01, *** *p* < 0.001 by two-way ANOVA with Tukey’s multiple comparisons test, *n* = 3 in each group. ns: no significance.

Hyaluronan is not only a major polysaccharide component of the extracellular matrix, but also plays a key role in the regulation of many cellular processes and in the organisation of tissue architecture [16]. Our previous results have suggested that 4MU can be considered as a potential treatment for CNS pathologies [6,8]. Here, we investigated the potential adverse effects induced by long-term systemic administration of 4MU at a dose of 1.2 g/kg/day. Immunohistochemical analysis of HABP and ACAN or CS-56 were performed in brain, spinal cord, spleen, liver and kidney (Figure 1 and Figure 2). We observed significant downregulations of staining intensity in the tissues, confirming the effect of 4MU in HA and CS reduction.

4-MU treatment in healthy animals decreased the intensity of both ACAN- and HABP-positive signals in the brain by almost 50% (ACAN: 46.16 ± 6.23%, *n* = 3; *p* = 0.0128; HABP: 44.26 ± 5.71%, *n* = 3, *p* = 0.0141; Figure 1A–H). After 9 weeks of wash-out period, the intensity did not return to more than 83% (ACAN: 79.1 ± 2.02%, *n* = 3; *p* = 0.1287; HABP: 72.9 ± 4.07%, *n* = 3, *p* = 0.0956; Figure 1A–D). In the spinal cord, there was an even stronger decrease in the fluorescent signal after 4-MU treatment (ACAN: 42.35 ± 4.62%, *n* = 3; *p* = 0.0012; HABP: 63.26 ± 4.07%, *n* = 3, *p* = 0.00015; Figure 1E–H). After 9 weeks of wash-out, the level of aggrecan signal returned to almost 90% (ACAN: 89.7 ± 2.08%, *n* = 3; *p* = 0.2911). In contrast to the ACAN-positive signal, the HA level in the spinal cord returned to the original values (HABP: 45.29 ± 11.19%, *n* = 3, *p* = 0.9932; Figure 1E–H). Aggrecan was chosen as one of the markers of PNNs. In order to stain for CSPGs in other organs, we decided to replace aggrecan with CS-56 for visualisation in kidney, spleen and liver sections. 4-MU treatment demonstrates different effect in organs in healthy animals. In the liver, our results indicate a significantly reduced CS-56-positive signal (25.56 ± 1.13%, *n* = 3; *p* = 0.0173, Figure 2A,D,E), but the HABP-positive signal had no significant difference even after 4-MU treatment.

In the spleen, we did not observe any significant changes between any of the three groups (Figure 2B,D,E). In the kidney, we observed an increase in the fluorescence intensity of CS-56 (31.23 ± 7.64%, *n* = 3; *p* = 0.0018), and a decrease in HABP fluorescence intensity (44.88 ± 4.08%, *n* = 3, *p* = 0016; Figure 2C–E). Following 9 weeks of wash-out period, the CS-56-positive signal in the liver remained low compared to healthy controls (*p* = 0.0009), in contrast to the kidney samples, where the CS-56-postive signal remained high after the wash-out period (*p* = 0.0247). 

### 2.2. Neither Haematological nor Biochemical Parameters Indicate Adverse effects Caused by Long-Term Administration of 4MU at the Same Dose

**Figure 3:**
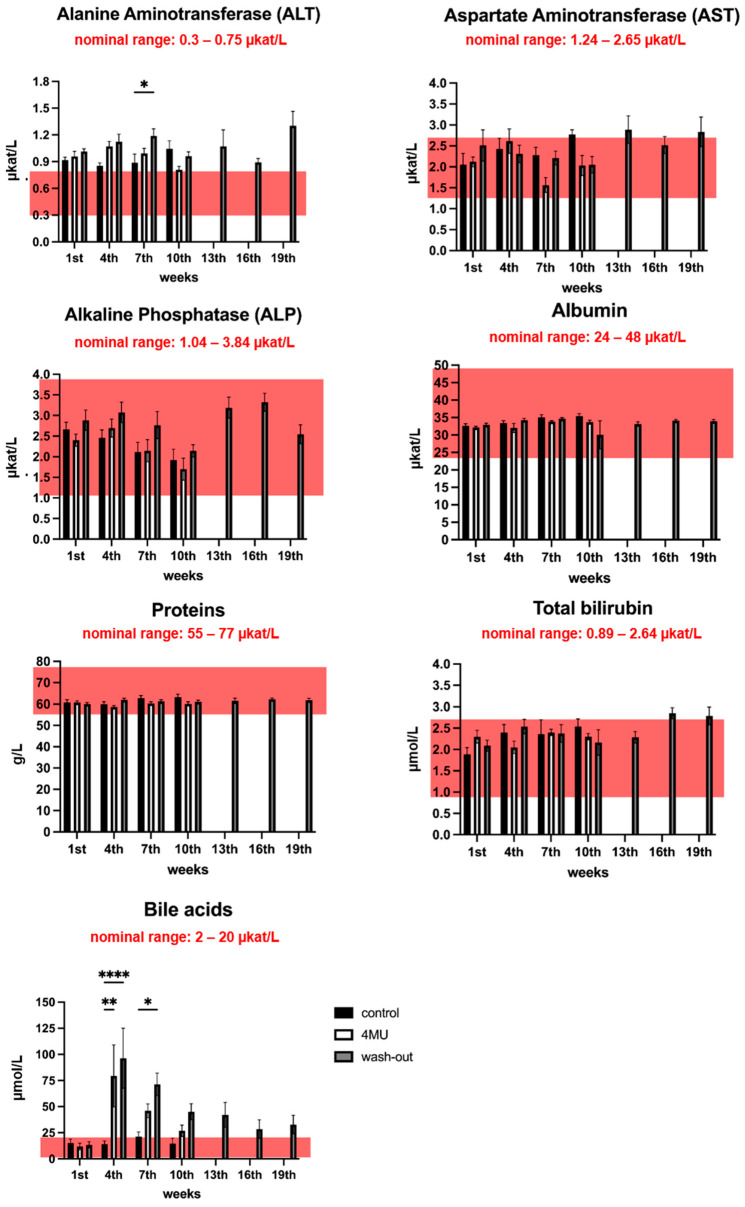
Liver function tests do not indicate any liver damage after long-term 4MU treatment at 1.2 g/kg/day dose. The pink box in each graph indicates the nominal range of the corresponding test. * *p* < 0.05, ** *p* < 0.01, **** *p* < 0.0001 by two-way *ANOVA* with Tukey’s multiple comparisons test, *n* = 5 to 7.

We then investigated whether long-term treatment with 1.2 g/kg/day 4MU affects any of the haematological parameters, including leukocytes, haemoglobin, haematocrit, erythrocytes (concentration, mean volume and colorant), red cell distribution width, thrombocytes, reticulocytes, neutrophils, lymphocytes, monocytes, eosinophils, basophils and neutrophils (Figure 3). The data are presented in Supplementary Materials, Table S2. Haematological parameters from all three groups were compared with reference values (“Clinical Laboratory Parameters for Crl:Wi (Han) rats”, Charles River Laboratories International, 2008). All parameters were within the reference range, except for absolute and relative monocyte counts, where data fell outside the reference range. In the case of monocytes, the numbers increased in all three groups without any significant difference. 

Next, we tested the biochemical parameters of blood and blood serum. The following parameters were monitored: sodium, potassium, calcium, phosphorus, creatinine, urea (Table S3) and markers associated with liver injury (total bilirubin, bile acids, AST, ALT, ALP) (Figure 3). Since almost all drug classes can cause a drug-induced liver injury [17], we focused primarily on liver function tests to monitor potential liver injury. In week 7 of 4MU treatment, we observed significant differences in the measured ALT level between the wash-out group (1.189 ± 0.080 μkat/L) and placebo (0.886 ± 0.097 μkat/L). Moreover, we observed slightly higher ALT enzyme levels (mean 1.005 ± 0.133 μkat/L) than the reported reference value (0.3–0.75 μkat/L) throughout the experiment.

4MU has been shown to increase bile acid secretion and is used in biliary stasis [18]. We thus checked the concentration of bile acid after 4MU treatment. We observed a significantly higher level of bile acids in the blood at 4 weeks post treatment when compared to control (4MU 79.36 ± 29.67 μmol/L, wash-out 96.36 ± 28.69 μmol/L vs. control 14.15 ± 3.11 μmol/L; *p* = 0.0014 and *p* < 0.0001, respectively). In week 7, the bile acid level remains high in the treated group, although significance is only observed in the wash-out group, reflecting a potential physiological adaptation to 4MU from long-term treatment (4MU 46.07 μmol/L, wash-out 71.37 ± 10.78 μmol/L vs. 21.296 ± 12.803 μmol/L; *p* = 0.38 and *p* = 0.0235, respectively). Our data revealed no other significant differences between the groups in the remaining haematological markers of liver injury.

The results suggest that long-term 4MU treatment did not induce any serious adverse changes in haematological or biochemical parameters in blood.

The levels of serum glucose, cholesterol, triacyl glycerides and iron were also evaluated in blood serum. The data are presented in Supplementary Materials, Table S4. No renal impairment was observed after 10 weeks of treatment with 4MU at a dose of 1.2 g/kg/day.

**Figure 4:**
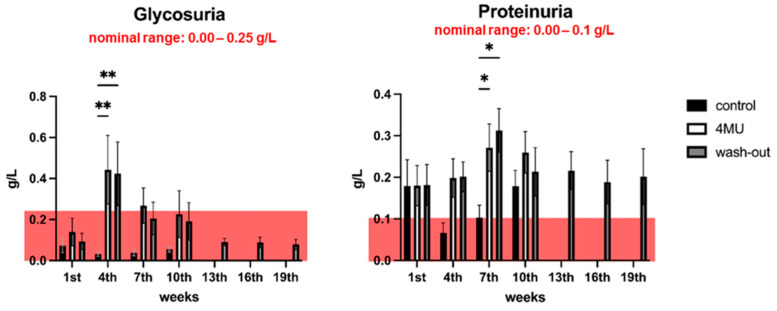
Glycosuria and proteinuria show no severe kidney damage after 1.2 g/kg/day of 4MU-treatment. The bar graph shows the level of urinary glucose changes (**A**) and urinary protein changes (**B**) during the 4MU treatment. The pink box in each graph indicates the nominal range of the corresponding test. Data are expressed as a mean ± SEM; * *p* < 0.05, ** *p* < 0.01 by two-way *ANOVA* with Tukey’s multiple comparisons test, *n* = 7.

Based on the histochemical changes in the kidneys, glucose and protein levels were analysed every three weeks (Table S4). Glycosuria, an abnormal amount of glucose present in the urine, is usually associated with impaired kidney filtration. Glycosuria was observed in the fourth week in the 4MU-treated group (0.443 ± 0.167 g/L) and in the wash-out group (0.424 ± 0.155 g/L) compared to the placebo group (0.032 ± 0.011 g/L) (Figure 4A). In weeks 7 and 10, the urinary glucose results remained slightly increased in the 4MU-treated and wash-out groups compared to the reference values, but there was no significant difference from the placebo group. During the wash-out period, urine glucose levels returned to the normal physiological range (Figure 4A).

Kidney damage is often associated with elevated urinary protein levels, referred to as proteinuria. Proteins outside normal physiological values were observed in the 4MU-treated and wash-out groups (Figure 4B), as well as in the placebo group in week 1 (0.179 ± 0.064 g/L) and week 10 (0.179 ± 0.038 g/L) of the sampling period. Only in week 7, there was a significant difference in the placebo group (0.103 ± 0.030 g/L) compared to the 4MU-treated (0.271 ± 0.057 g/L) and wash-out (0.313 ± 0.052 g/L) groups. During the wash-out period, protein levels did not return to physiologically normal values (Figure 4B).

**Figure 5:**
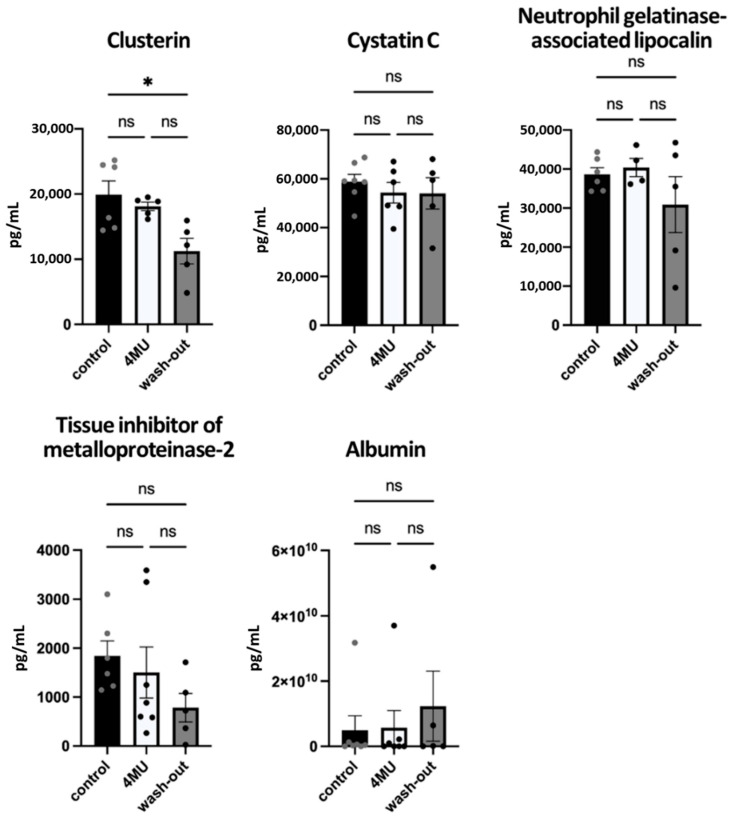
Urinary biomarkers used to assess the nephrotoxicity. Bar graphs show the level of urinary markers of renal injury. Data are expressed as a mean ± SEM; * *p* < 0.05 by one-way *ANOVA* with Tukey’s multiple comparisons test, *n* = 4 to 7. ns: no significance.

To further investigate the effect of drug metabolism on renal injury, the Rat Kidney Toxicity 5-Plex ProcartaPlex Panel 2 (Invitrogen, #EPX050-30125-901) was used. Five urinary biomarkers were chosen to assess nephrotoxicity (Figure 5): clusterin, cystatin C (Cys-C), neutrophil gelatinase-associated lipocalin (NGAL), urinary tissue inhibitor of metalloproteinases-1 (TIMP-1) and albumin. Clusterin is usually associated with tubulointerstitial renal lesions, and drug-induced damage is manifested by reduced gene and protein expression. Cys-C is filtered, completely reabsorbed and catabolised in the proximal tubule under physiological conditions. In acute kidney injury, the urinary concentration of Cys-C increases. NGAL, an iron-transporting protein, increases its urinary excretion after nephrotoxic and ischemic insults, and thus is among the biomarkers of acute kidney injury [19]. TIMP-1 levels are low in healthy kidneys, but have been shown to increase significantly in models of kidney diseases and are also associated with the extent of fibrosis [20]. The last marker studied was albumin, a well-established diagnostic and prognostic marker for assessing the degree of glomerular disease severity in the progression of chronic kidney disease. Our results showed significantly reduced levels of clusterin in the wash-out group (12.861 ± 1442 pg/mL) compared to the placebo (19.903 ± 2121 pg/mL) and the 4MU-treated group (18.115 ± 647 pg/mL). No significant differences were observed between groups in Cys-C, NGAL, TIMP-1 and albumin levels. 

### 2.3. 1.2 g/kg/Day Dose of 4MU Increases Levels of IFN-γ, IL10 and IL12p70 in Blood Serum after 10 Weeks of Daily Administration but Values Returned Back to Control Levels during the Wash-Out Period

As there is evidence showing that HA regulates cytokine release [7], we investigated whether 4MU-mediated inhibition of HA synthesis would affect the process. The Complete 14-Plex Rat ProcartaPlex™ Panel (Invitrogen, #EPX140-30120-901) was used to measure the concentration of fourteen cytokines (IFN-γ, IL10, IL12p70, IL13, IL2, IL17a, IL4, IL5, IL6, IL1α, IL1β, TNF-α, GM-CSF and G-CSF) in blood serum. However, we were only able to detect six of them. The other eight markers were either negative or below the detection limit of the kit.

**Figure 6:**
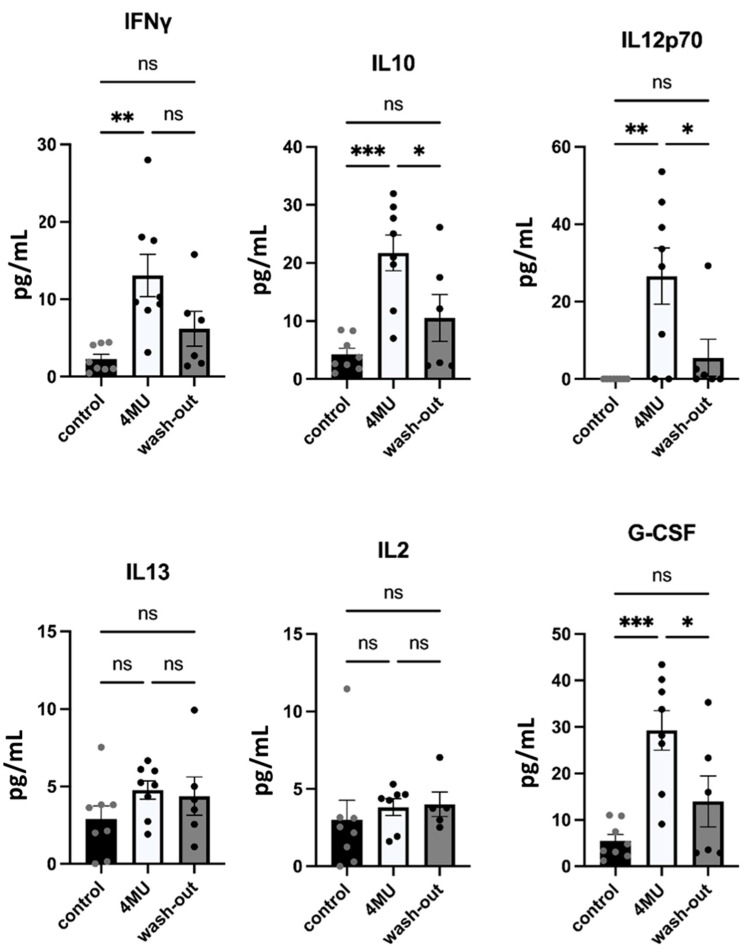
Serological levels of cytokines and interleukins after long-term 4MU treatment. Bar graphs show the level of detected cytokines and interleukins. Data are expressed as a mean ± SEM; * *p* < 0.05, ** *p* < 0.01, *** *p* < 0.001 by one-way ANOVA with Tukey’s multiple comparisons test, *n* = 5–8. ns: no significance.

Our results indicate significantly increased levels of IFN-γ, IL10, IL12p70, and G-CSF in 4MU-treated animals when compared to the control placebo group, with a decrease back to the control values in the wash-out group. Furthermore, after the wash-out period, the values of all markers affected by 4MU treatment returned to those of healthy controls. IL13 and IL2 were not significantly altered compared to the control group (Figure 6).

### 2.4. 1.2 g/kg/Day Dose of 4MU Does Not Affect Behavioural Performance of the Experimental Animals

**Figure 7:**
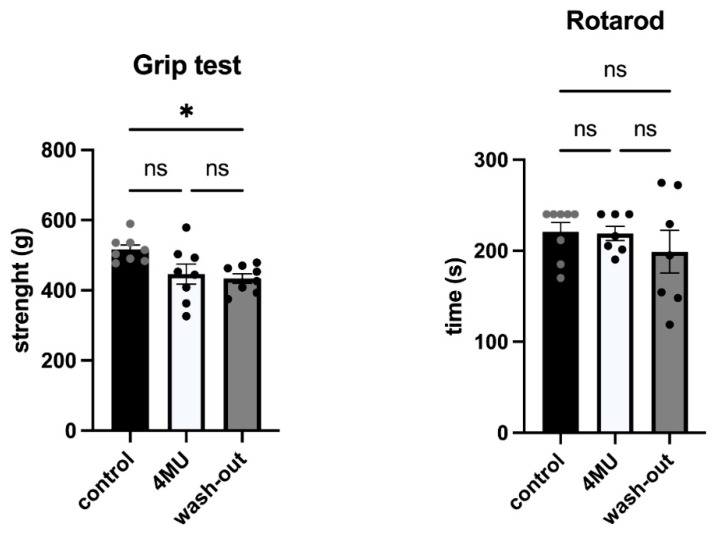
The results between placebo, 4MU-treated and wash-out groups did not show any changes in motor functions after 10 weeks of 4MU treatment, but significant difference was observed in the strength of the forelimbs between placebo and wash-out groups. Different color dots are used for easy identification of each data points. Data are expressed as mean ± SEM; * *p* < 0.05 by one-way *ANOVA* with Tukey’s multiple comparisons test. ns: no significance.

Various behavioural tests were used to determine the possible behavioural changes that 4MU might have on locomotor functions in rats (Figure 7). Grip strength test was used to assess neuromuscular functions by determining the maximum force exhibited by the animal. Animals in the wash-out group had significantly reduced isometric forelimb contraction strength compared to the control placebo group, but the reduction was mild. Rotarod was used to assess motor function and forelimb-hindlimb coordination. No significant changes were found between groups. 

### 2.5. 1.2 g/kg/Day Dose of 4MU Increases the Relative Number of Proerythroblasts in Bone Marrow, but Returned to Normal after the Wash-Out Period

**Figure 8:**
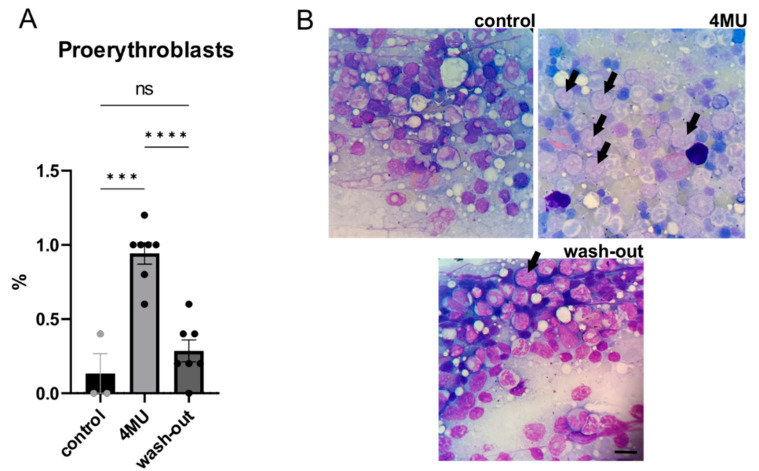
Relative number of proerythroblasts in the bone marrow. (**A**) shows the bar graph comparing the relative number of proerythroblasts in 1000 cells in bone marrow per the smear; (**B**) shows representative images of the bone marrow smear. Black arrows show the proerythroblasts; scale bar: 20 µm; *n* = 8; *** *p* < 0.01; **** *p* < 0.001 by one-way *ANOVA* with Tukey’s multiple comparisons test. ns: no significance.

HA plays a key role in the highly complex regulatory network of the hematopoietic microenvironment [21]. Accordingly, we examined bone marrow smears to assess the effect of 4MU treatment on haematopoiesis. The results showed significant changes in the percentage of proerythroblasts in the smear. Proerythroblasts, the earliest of the four developmental stages of the normoblast, were significantly higher in the 4MU-treated group compared to the placebo and wash-out groups. HA forms a regulatory element in the hematopoietic microenvironment [21], suggesting that 4MU-mediated HA inhibition disrupts this microenvironment, which is crucial for successful haemopoiesis and leads to a significant increase in the number of proerythroblasts per 1000 cells in 4MU-treated animals (Figure 8).

### 2.6. Long-Term 4MU Treatment at the Current Dose Does Not Affect the Biomechanical Properties of Tendons and Skin

There are several studies evaluating the contribution of HA to tendon growth and maturation [22] and skin turgor [23]. To evaluate whether 4MU-mediated HA inhibition affects skin or/and tendon integrity, biomechanical properties were measured (Table 1). The mechanical properties of rat skin were evaluated using a uniaxial tensile test. The results obtained from the mechanical tests showed no statistical differences (*p* = 0.05) between the Young’s modulus of elasticity in the skin tension, determined at two values of longitudinal relative strain of 5% and 10%, for the placebo control group (Young’s tensile modulus of 9.326 (7.57–10.97) MPa at 5%, and 16.43 (10.79–17.37) MPa at 10%), the 4MU-treated group (13.57 (10.45–16.47) MPa at 5%, and 16.06 (8.82–17.13) MPa at 10%) and the wash-out group (10.43 (8.43–12.27) MPa, and 16.79 (12.29–20.20) MPa at 10%). 

The mechanical properties of the tendons were evaluated by uniaxial tensile test. The results showed that there was no statistical difference between the tensile strength of the Achilles tendon and ultimate strength value for the placebo group, the 4MU-treated group and the wash-out group (*p* = 0.05). The ultimate strength value from the tensile test was 20.11 ± 2.673 N for the placebo control group, 14.75 ± 1.746 N for the 4MU-treated group and 23.56 ± 3.708 N for the wash-out group. The tensile strength of the Achilles tendon reached 23.2 ± 3.015 in the placebo group, 22.51 ± 3.531 in the 4MU-treated group and 38.83 ± 7.662 in the wash-out group. The tensile strength value of the wash-out group increased by up to 178% of that of the placebo group, but this difference was not statistically significant (*p* = 0.05). 

## 3. Discussion

In this study, we investigated the systemic effects after a 10-week 4MU treatment followed by an 8-week wash-out. The results showed that the levels of HA and CSPGs were reduced. Corresponding with the 4MU-mediated choleretic activity, there was a significant increase in bile acids. There was also an increase in blood sugars and proteins a few weeks after 4MU administration. In addition, the level of interleukins IL10, IL12p70 and IFN gamma significantly increased after 10 weeks of 4MU treatment. However, no significant differences were found between control-treated and 4MU-treated animals after the 9-week wash-out period. The results suggest that long-term 4MU treatment is well-tolerated and does not induce adverse effects on normal physiology. 

4MU was previously identified as an inhibitor of HA synthesis. HA is a non-sulphated glycosaminoglycan composed of disaccharide repeats of D-glucuronic acid (GlcA) and N-acetyl-D-glucosamine. 4MU inhibits HA synthesis by depleting UDP-glucuronic acid (UDP-GlcA), which is an essential substrate for HA, via glucuronidation of 4MU [24,25,26]. In addition, this leads to a reduction in HAS mRNA levels [27]. Here, we report that 4MU also reduces the synthesis of CS, which shares the same monosaccharide, GlcA, as the key basic component. As CS is a key inhibitory molecule for neural regeneration and plasticity, our results suggest that 4MU could potentially be applicable for a novel non-invasive treatment for nervous system conditions. 

In our experiments, we used a systemic route of administration and assumed that the whole body of the animal would be affected. In order to investigate the systemic effect of 4MU treatment at a dose of 1.2 kg/g/day, we decided to map the pathophysiological changes in more detail. Our results showed that long-term 4MU administration did not cause irreversible adverse effects.

**Figure 9:**
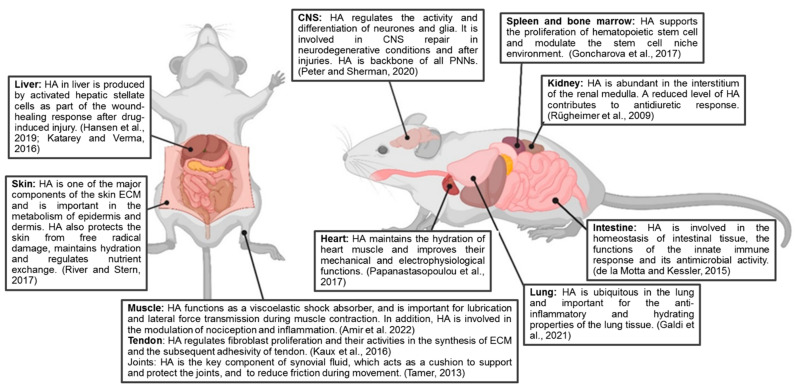
Schematic representation showing the diverse biological functions which HA is involved. Created with BioRender.com, Refs. [21,29,32,33,34,35,36,37,38,39,40].

HA is evolutionary conserved and abundantly expressed throughout the body. As a simple linear polysaccharide, HA exhibits a wide range of biological functions. HA interacts with various molecules, thereby maintaining tissue homeostasis and organising the structure of the ECM. The exceptional biophysical and biomechanical properties of HA contribute to tissue hydration, mediate the diffusion of solutes through the extracellular space and maintain tissue lubrication. Binding of HA to cell surface receptors activates numerous signalling pathways that regulate cell function, tissue development, inflammation progression, wound healing responses and tumour biology, as reviewed in [28] (Figure 9). In the CNS, HA regulates neuronal and glial cell differentiation, neuronal activity and plays a role in neurodegenerative diseases and CNS injuries (summarised in [29]). HA is also associated with the dense ECM structure that enwraps certain types of neurons in the brain and the spinal cord, called PNNs, where it forms the PNN backbone [30]. After treatment with 4MU at the current dose, we observed downregulation of HA and PNNs in the brain and spinal cord. Indeed, we have previously observed that 4MU treatment enhances memory retention in adult mice [5]. However, PNNs have recently been shown to be involved in several psychiatric disorders such as schizophrenia, autism spectrum disorders and mood disorders [31]. This suggests that pharmacological dose assessment is only the first step in evaluating the effect of 4MU, and behavioural studies addressing the potential development of psychiatric disorders following PNN downregulation will be needed for future clinical relevance.

Outside the CNS, HA also regulates the physiological functions of other organs. In the lungs, HA is mainly present in the lung connective tissue [41] and is involved in the formation of viscous gel, which plays a key role in tissue homeostasis, in the regulation of fluid balance in the lung interstitium and their biomechanical integrity [42]. We did not observe any pathological changes in histology after treatment with 4MU at the current dose, suggesting that the current dose administered for 10 months does not lead to structural changes in lung tissue.

HA is abundant in the heart, where it is involved in cardiac physiological functions [43] as well as in pathological conditions [44]. Although HA is one of the key molecules in the heart, improving electrophysiological and mechanical functions, the reduction in HA after treatment with 4MU in our study is not indicative of pathological changes in the heart tissue. 

HA in the spleen is important for CD-44-mediated progenitor cell adhesion. When the affinity for CD-44 is dysregulated following impaired HA synthesis, this is accompanied by morphological changes in the spleen such as striking enlargement [45] and increased hyaluronidase activity, causing impaired GAG metabolism in the connective tissue [46]. We did not observe any significant changes in splenic HA distribution and/or any pathology between the groups in the histological examination of the spleen. On the contrary, we observed significant changes in bone marrow smear after long-term 4MU treatment. Bone marrow is composed of many cell types, such as bone-forming osteoblasts and hematopoietic stem cells. HA forms an essential element in the regulatory network of haematopoiesis. In the hematopoietic microenvironment, HA is actively involved in the regulation of cytokine and chemokine production and cell motility [47]. In our experiment, we observed a significantly high proportion of proerythroblasts in the 4MU-treated group compared with the placebo and wash-out groups. Proerythroblasts are the earliest stage cells out of the four in the development of normoblasts. Thus, they demonstrate the important role that ECM plays in haemopoiesis by allowing hematopoietic cells to adhere to the marrow stroma and also bind growth factors controlling haemopoiesis [48]. The close contact facilitated by the ECM has been shown to be a controlling factor required for successful haematopoiesis [21]. This suggests that 4MU-mediated inhibition of HA synthesis results in the loss of some of the cell–cell interactions necessary for successful haemopoiesis and leads to a significantly higher relative number of proerythroblasts per 1000 cells in 4MU-treated rats compared with the other groups.

The liver has been shown to be the most important organ involved in the synthesis and degradation of HA [49], and at the same time, the key organ for detoxification. Glucuronidation is one of the major mechanisms for drug detoxification and requires UDP-GlcA, the key substrate for HA synthesis. 4MU administered to the body will be removed through this metabolism in the liver. To investigate the effect of 4MU on the liver itself, liver function tests were performed. We monitored two of the enzymes: ALT and AST. We observed slightly higher levels of ALT in all three groups throughout the experiment when compared to the reference range. In the seventh week, we also observed significantly increased ALT in the wash-out group compared to the placebo group. It has been shown that liver enzyme levels can change even under normal physiological conditions [50] or as a result of intense physical activity [51]. In the case of AST [52], our data showed that AST levels were within the physiological range throughout the duration of the experiment.

In addition to liver enzymes, we also examined albumin, total protein, bilirubin and bile acids in the blood throughout the experiment. Albumin and the total protein levels were within the physiological norms throughout the experiment. The last marker monitored was bilirubin, a breakdown product of red blood cells, and elevated levels may also indicate liver damage. Our biochemical results together with pathological examination revealed no liver damage after long-term 4MU treatment at the current dose, suggesting that 4MU at this dose does not lead to drug-induced damage even after 10 weeks of daily administration. 

In the gut, HA facilitates nutrient and water absorption as well as the continuous interaction with GAG-rich (glycosaminoglycans) interstitial ECM [53]. During pathological conditions when nutrient and water intake are compromised, HA distribution is altered [54]. Under pathophysiological conditions where HA synthesis is disrupted, increased bacterial translocation and dysbiosis occurs, as well as permeabilisation mediated by disrupted tight junctions. In addition, there is recruitment of mononuclear cells and increased adhesion in lamina propria. We did not observe any pathophysiological changes in the intestine or other complications related to intestinal damage following long-term 4MU treatment. 

Over the past decades, HA has emerged as a key player in nephrology and urology studies involving ECM organisation, inflammation, regeneration, as well as pathological processes [55]. With reference to 4MU, it has already been shown that HA inhibition could be protective against renal ischaemia reperfusion injury [56]. Despite the fact that we observed a loss of HA when staining renal sections for HABP, we did not observe any pathological changes after 10 weeks of 4MU treatment. Urinary biochemical evaluation revealed glycosuria in week 4 and proteinuria in week 7 in 4MU-treated animals. Markers of nephrotoxicity did not indicate any evidence of kidney damage. The only significant difference was observed in urinary clusterin levels. A significant reduction in clusterin levels was observed in the wash-out group compared to the placebo, which may be related to the age of the animals rather than to the 4MU treatment itself. 

In mammals, about 27% of total HA is expressed in the skeleton and connective tissue, whereas only about 10% is expressed in muscle [57]. Studies have already shown that HA is mainly concentrated in joint synovial fluid, skin and muscle connective tissue [58], fascia and loose connective tissue [59]. HA plays a key role in lubrication as well as in lateral force transmission during muscle contraction [60]. To test muscle contraction and muscle strength, the animals were behaviourally tested on rotarod and grip strength. We did not observe any significant difference between the placebo and the 4MU-treated group that would indicate impairment of muscle contraction or strength. The significant difference observed between the placebo and wash-out groups in the grip strength test is likely related to age-related changes in the neuromuscular system [61]. Due to the high expression of HA in tendons, where it enhances fibroblast cellular activity [36], and in synovial fluid [37] between joints, which protects bone ends and provides support during movement, we also tested the biomechanical properties of tendons. Our results suggested no significant changes indicating an adverse effect of 1.2 g/kg/day 4MU.

HA was found to be responsible for skin moisture in the skin [62] and wound healing [63]. HA is synthetised by both dermal but also epidermal cells. Dermal HA, unlike epidermal HA, is primarily responsible for skin hydration. A decrease in epidermal HA is directly associated with skin ageing; on the other hand, dermal HA has been shown to remain constant with ageing [64]. Our results suggest that the 4MU treatment did not lead to downregulation of HA in the skin nor alter the biomechanical properties. 

HA is also an important player in the regulation of the immune response [65]. As part of the immune response, inflammation plays a key role in the body’s defence against pathogens. However, inappropriate activation of inflammatory processes can contribute to many pathological conditions [66]. IFN-γ and IL10 have been described in the context of hyaluronan synthesis, and both appear to inhibit chemokine gene expression by altering mRNA stability and transcription of MIP-1α and MIP-1β genes [67]. Furthermore, IL10 plays an important role as a regulator of HA synthesis in addition to its role in anti-inflammatory responses. IL10 binds to IL10R1 and IL10R2 to form a tetrameric complex and activates the JAK/STAT3 pathway. This pathway leads to phosphorylation and nuclear translocation of STAT3, which activates genes such as hyaluronan synthase (HAS) [68]. This could explain the significantly higher IL10 levels in 4MU-treated animals as an attempt by the organism to compensate for the 4MU-mediated HA inhibition.

## 4. Materials and Methods

### 4.1. Animals

Healthy 10-week-old female Wistar rats (*n* = 24,250 ± 30 g) were obtained from Janvier Labs (CS 4105 Le Genest Saint Isle; Saint Berthevin Cedex 53941 France). This study involved only female rats to reduce the potential interference from rapid weight gain observed in male rats in long-term study. Animals were assigned randomly into 3 groups (*n* = 8/group): placebo group, treated group (4MU) and wash-out group. For the whole duration of the experiment, the rats were housed in pairs under a 12 h light/dark cycle with standard conditions. Rats received tap water and food *ad libitum*. 

After 10 weeks of the 4MU treatment, animals from placebo and 4MU-treated groups were intraperitoneally anesthetised with a lethal dose of ketamine (100 mg/kg) and xylazine (20 mg/kg). Animals were then transcardially perfused with phosphate-buffer saline (PBS) and 4% paraformaldehyde in PBS. The wash-out group was sacrificed after another 9 weeks.

The experiments were approved by the ethical committee of the Institute of Experimental medicine of ASCR and performed in accordance with Law No. 77/2004 of the Czech Republic. Based on previous studies, the number of animals was statistically optimised for each particular experiment to achieve their reduction according to the European Commission Directive 2010/63/EU.

### 4.2. Drug Dosage

Rats were fed *ad libitum* with chocolate-flavoured chow (Sniff GmbH, Soest, Germany) (placebo) or with chocolate-flavoured pellets containing 4MU (4-methylumbelliferone, DbPharma France) at a dose of 1.2 g/kg/day ± 10% of 4MU (i.e., 2.5% *w*/*w* dose in the chow). The 10% variation represents the variability of food intake between rats, and also between individual weeks. The animals were treated for 10 weeks. The wash-out group was fed with 4MU for 10 weeks, after which they were left untreated for a further 9 weeks. Rats and chow consumed were weighed weekly throughout the 4MU treatment. While each rat was weighed individually, the chow was then weighed from the food hopper and an average of the chow consumption was calculated.

### 4.3. Haematology and Biochemistry

Animals were anesthetised with an inhalant mixture of isoflurane and air (3 *v*/*v*% isoflurane, AbbVie, Chicago, IL, USA), with an inflow of 300 mL/min, and kept under anaesthesia for the entire duration of the blood sample collection. Blood was collected from the retro-orbital venous sinus by inserting an autoclaved Pasteur pipette tip into the medial canthus of the eye under the nictitating membrane. The pipette tip was positioned between the globe and the bony orbit of the eye. Blood was collected every 3 weeks (in the 1st, 4th, 7th, 10th week of the experiment in all three groups and in weeks 13, 16, 19 in the wash-out group). Blood samples were collected into BD Microtainer^®^ blood collection tubes. Blood samples for haematological analysis were collected in anticoagulant tubes containing K2EDTA (#365974), and the following parameters were assessed: red blood cells count, haemoglobin, haematocrit, white blood cell count and relative number of white blood cells. Blood samples for biochemical analysis were collected in a serum-separating tube (#365963), and the following serum parameters were determined: sodium, potassium, calcium, phosphorus, urea, creatinine and total bilirubin. Results were evaluated by Synlab (Munich, Germany).

While the animal was still under anaesthesia, urine was collected. The rat was held over a Petri dish, and by manual pressure on the lower abdominal area, urine was squeezed out of the bladder. The urine was collected with a syringe into an Eppendorf tube (1.5 mL). In every sample, levels of glucose and proteins were assessed. Urine was evaluated by Synlab (Munich, Germany) as blood samples.

### 4.4. Immunohistochemistry

Brain and spinal cord tissue were embedded in O.C.T. compound (VWR) and sectioned in a frozen block of tissue into 40 μm thick slices for further immunohistological staining. At room temperature (RT), free-floating 40 µm sections were washed 3 times for 10 min each in 1x PBS in order to remove cryoprotectant (ethylene glycol:glycerol:PBS; 3:3:4) residue. Tissue was then permeabilisated with 0.5% triton in PBS for 2 h. Then, the Avidin/Biotin blocking kit (Abcam; ab64212) was used to decrease non-specific signal by blocking the endogenous biotin. Tissue was then blocked in the solution containing ChemiBLOCKER (Millipore, Darmstadt, Germany #2170; 1:10), 0.3 M glycine, 0.2% triton, PBS) for another 2 h. The sections were then transferred to co-incubate at 4 °C in immunoblockade containing the following antibodies: biotin-conjugated HABP (b-HABP; Amsbio #AMS.HKD-BC41; 1:200; 48 h); anti-ACAN (rabbit; Sigma, Darmstadt, Germany #AB1031; 1:300; 48 h); anti-CS-56 (mouse; Sigma #C8035; 1:200; 48 h). To visualise each primary antibody staining, the tissue was then co-incubated with the appropriate species of fluorescent-conjugated secondary antibodies (Invitrogen, Darmstadt, Germany, #S32354; #S21374; #A-11005; #A-11037; 1:300; 6 h; RT). Then, DAPI (4’,6-diamidino-2-phenylindole, dihydrochloride) in 0.2% triton in PBS was used (Invitrogen #D1306; 1:2000; 10 min). Samples were mounted in Mowiol^®^ mounting media. Samples were observed and captured with Zeiss LSM 880 Airyscan microscope (Carl Zeiss AG; Oberkochen, Germany) to evaluate the HA and ACAN/CSPGs changes in organ sections. Three replicate sections were imaged for all organs on a Zeiss LSM 880 confocal microscope and analysed in FIJI^TM^ (NIH ImageJ). Image analysis parameters were kept constant among all samples.

### 4.5. Proteomics

For evaluation of kidney toxicity, proteins in urine were evaluated using the Kidney Toxicity 5-Plex Rat ProcartaPlex™ Panel 2 (Invitrogen # EPX050-30125-901). Prior to the transcardial perfusion, urine samples were manually collected from each rat, then stored in −80 °C. The Rat Kidney Toxicity 5-Plex ProcartaPlex Panel 2 allows the analysis of 5 protein targets, namely albumin, cystatin, clusterin (Apo-J), NGAL and TIMP-1, in a single well using Luminex xMAP technology to determine the potential nephrotoxicity during drug studies. Samples were measured according to the manufacturer’s protocol.

For evaluation of T helper response, the Th Complete 14-Plex Rat ProcartaPlex™ Panel (Invitrogen # EPX140-30120-901) was used. This panel allows the analysis of 14 protein targets in a single well using Luminex xMAP technology. Targets are IL-1α; G-CSF; IL-10; IL-17A; IL-1β; IL-6; TNFα; IL-4; GM-CSF; IFNγ; IL-2; IL-5; IL-13; IL-12p70. Samples were measured according to the manufacturer’s protocol on Bio-Plex 200 Systems (Bio-Rad, Hercules, CA, USA).

### 4.6. Behavioural Tests

To assess the behavioural effect of 4MU, 2 tests were selected. The rotarod was used to assess motor performance and motor coordination and the forelimb grip strength test aimed to test neuromuscular functions by determining the maximal force developed by a rat. Both tests were used to investigate whether 4MU has any effect on motor functions and/or joint mobility, and were performed after 10 weeks of 4MU treatment in all three groups and 9 weeks after 4MU treatment in the wash-out group. Prior to the rotarod (ROTA-ROD 47700, UGO BASILE S.R.I.) training session, rats were habituated to remain on a stationary drum for 60 s and pretrained at 5 rpm for 120 s. In the testing phase, animals were tested for 300 s with increasing acceleration from 5 to 10 rpm for the first 180 s and then for additional 120 s with acceleration of 10 rpm on three different days following training. The time at which the rat fell off the rotating bar was recorded. For the forelimb grip strength, an instrument (BSGT2S; Harvard Apparatus, Holliston, MA, USA) was used. The researcher pulled horizontally on the tail of the rat, which was gripping a metal grid attached to the monitoring device. The force applied to the grid before the rat lost its grip was recorded as the maximal muscle force. The test was repeated on three different days over the course of one week. Data from 4-MU treated group and wash-out group were collected independently. 

### 4.7. Histopathological Evaluation

For histopathological evaluation, spleen, liver, kidney, heart and small intestine were collected after transcardial perfusion and fixed for 24 h in 4% paraformaldehyde in 0.1 M PBS. Formalin-fixed paraffin-embedded (FFPE) samples were cut into 5 µm thin slices on a Leica RM2255 (Leica Biosystems Nussloch GmbH, Nußloch, Germany) microtome, placed on standard slides (Bammed s.r.o, Czech Republic) and stained with haematoxylin–eosin (DiaPath, Martinengo, Italy).

### 4.8. Bone Marrow Evaluation

Bone marrow was collected from tibia. Tibias were cleaned of fat, muscles and tendons. Then, the tibial heads were cut off with scissors and the bone marrow was flushed out of the bone with a 10 mL syringe. The bone marrow suspension was placed on one side of a microscope slide, gently spread with a second slide to form a thin film on the slide and fixed with methanol. Cells were stained using Pappenheim panoptic staining, a combination of May–Grünwald staining and Giemsa staining. This staining method allows the visualisation of basophilic, neutrophilic and eosinophilic blood structures. The May–Grünwald solution contains two dyes, eosin and methylene blue. We first allowed the methanol-fixed bone marrow smear to dry. May–Grünwald solution was then added (5 min). Next, distilled water was used to remove the May–Grünwald solution (5 min). Following this step, Romanowsky–Giemsa stain was added (10 min) and then washed with distilled water. The microscope slides were then allowed to dry, and the blood smear was observed with a microscope (Olympus CX41, Shinjuku City, Tokyo, Japan) using 100× objective lens and immersion oil. The relative number of different elements in the smears was then determined by counting and classifying 1000 cells in the bone marrow per smear.

### 4.9. Biomechanical Testing

#### 4.9.1. Conditions and Equipment

Tested tissues were frozen for preservation. After thawing, the specimens were hydrated in a physiological saline solution at 8 °C for a period of 24 h before the experiment. The specimens were left at room temperature (23 ± 2 °C) for 2 h before the test. The MTS Mini Bionix 858.02 biomechanical servohydraulic testing system (MTS, Minnesota, USA) with load cell with a measuring range of 0–500 N (detector error max. 0.05%, e.g., 0.08 N error when 160 N is applied) was used for the three-point bending test, as well as the tensile test of skin and the tensile test of tendons. 

#### 4.9.2. Tensile Tests of Skin

The mechanical properties were evaluated by uniaxial tensile tests of rectangular strips of skin (width of the samples was 8 mm; the thickness was 0.8–1.0 mm). The experimental methodology was based on the determination of the ultimate force during the tensile test and the modulus of elasticity of the skin at a defined deformation. The evaluation parameters were Young’ tensile modulus *E* determined at two values of longitudinal relative deformation, 5% (E_5_) and 10% (E_10_). These values were obtained as the tangent direction of the linear region of the load curve of the dependence of the true stress on the relative deformation. The biomechanical servohydraulic testing system MTS Mini Bionix 858.02 and pneumatic jaws were used for the tensile test. Using the contrast marks on the surface of the specimens, the video extensometer automatically determined the reference length and elongation of the specimens. The specimens were attached to the pneumatic jaws and loaded at a constant rate of 20.0 mm/min until destruction.

#### 4.9.3. Tensile Test of Tendons

An evaluation of the mechanical properties of the rat tendons was carried out by destructive tensile tests in order to evaluate their strength. Rat tendon specimens were delivered in a BTM (bone to muscle) disposition, i.e., the tendon was dissected on one side with part of the muscle and on the other side with part of the bone. This configuration allowed for a secure setting in the pneumatic jaws during testing. The muscle was frozen by soaking in liquid nitrogen for 5 s before testing each sample. Care was taken to ensure that the tendon did not freeze with the muscle. The MTS Mini Bionix 858.02 biomechanical servohydraulic testing system and pneumatic jaws were used for the tensile test. The tendons were fixed between two metal clamps mounted in pneumatic clamp grips, ensuring a constant downforce and tough, stable attachment of the samples during cross-sectional changes. The grips were equipped with diamond-like apexes. The samples were loaded at a constant speed of 10.0 mm/min until destruction. The evaluation parameters were the maximum force and maximum stress (i.e., strength), strength was calculated from the equation *σ_max_* = *F_max_*/*S* (MPa).

#### 4.9.4. Statistical Analysis of Data

Data are expressed as means ± SEM of *n* independent measurements. The significance of the difference between the means of two or three groups of data was evaluated using one-way ANOVA (HABP and ACAN intensity, behavioural test, proteomic, bone marrow evaluation, biomechanical testing) or two-way ANOVA (CS-56 and HABP intensity, haematological, serological and biochemical parameters, glycosuria and urinary proteins). A *p* value < 0.05 was considered statistically significant. Statistical analysis was performed with GraphPad Prism 9 software (version 9.3.0).

## 5. Conclusions

Our results show that 4MU at a dose of 1.2 g/kg/day after a 10-week treatment reduces whole-body HA levels; however, the current dose did not lead to any serious adverse effects. If any deviations from the reference values occurred, they normalised after a 9-week wash-out period in Wistar rats. Our findings indicate that 4MU could be considered as a long-term treatment at a dose of 1.2 g/kg/day. However, we did not address the possible behavioural changes causing PNN alteration in brain regions associated with psychiatric disorders such as schizophrenia, autism spectrum disorders and mood disorders. More detailed studies would be required to further characterise potential psychiatric adverse effects.

## Figures and Tables

**Table 1:** The biomechanical properties of skin and tendon in control, 4MU and wash-out groups.

**Skin**		
Young modulus E5 (5%)/MPa	control	9.326 ± 1.113
	4MU	13.57 ± 1.856
	wash-out	10.43 ± 1.240
Young modulus E10 (10%)/MPa	control	16.43 ± 2.910
	4MU	16.06 ± 2.702
	wash-out	16.79 ± 3.704
**Tendons**		
Maximum force Fmax/N	control	20.11 ± 2.673
	4MU	14.75 ± 1.746
	wash-out	23.56 ± 3.708
Maximum stress σmax/Nm^−2^	control	23.2 ± 3.015
	4MU	22.51 ± 3.531
	wash-out	38.83 ± 7.662

## Data Availability

Data are available in the Supplementary files.

## Supplementary Materials

The supporting information can be downloaded at: https://www.mdpi.com/article/10.3390/ijms24043799/s1.

## Abbreviations

4MU	4-methylumbelliferone
ACAN	Aggrecan
ALP	Alkaline phosphatase
ALT	Alanine transaminase
Apo-J	Apolipoprotein J
AST	Aspartate transaminase
BTM	Bone to muscle (deposition)
CNS	Central nervous system
CS-56	Anti-chondroitin sulphate antibody
CS	Chondroitin sulphate
CSPGs	Chondroitin sulphate proteoglycans
Cys	Cystatin C
DAPI	4’,6-diamidino-2-phenylindole, dihydrochloride
ECM	Extracellular matrix
FFPE	Formalin-fixed paraffin-embedded
GAG	Glycosaminoglycans
G-CSF	Granulocyte colony-stimulating factor
GlcA	D-glucuronic acid
GM-CSF	Granulocyte macrophage colony-stimulating factor
HA	Hyaluronan/hyaluronic acid
HAS	Hyaluronan synthase
HABP	Hyaluronan-binding protein
IFNγ	Interferon gamma
IL10	Interleukin 10
IL12p70	Interleukin 12p70
IL13	Interleukin 13
IL17a	Interleukin 17A
IL1α	Interleukin 1 alpha
IL1β	Interleukin 1 beta
IL2	Interleukin 2
IL4	Interleukin 4
IL5	Interleukin 5
IL6	Interleukin 6
IL10	Interleukin 10
JAK	Janus kinase
NGAL	Neutrophil gelatinase-associated lipocalin
PBS	Phosphate-buffered saline
PNNs	Perineuronal nets
RT	Room temperature
SCI	Spinal cord injury
STAT3	Signal transducer and activator of transcription 3
TIMP-1	Tissue inhibitor of metalloproteinases 1
TNFα	Tumour necrosis factor alpha
UDP-GlcA	UDP-glucuronic acid
