# Supplementary material for: 4-Methylumbeliferone Treatment at a Dose of 1.2 g/kg/Day Is Safe for Long-Term Usage in Rats
Source: Int J Mol Sci. 2023 Feb 14;24(4):3799. doi: 10.3390/ijms24043799 (PMC9959083; doi:10.3390/ijms24043799)
Supplement: Supplementary file 1 [file ijms-24-03799-s001.zip › Supplementary Table S1.pdf]

Supplementary Table S1 An overview of 4MU doses, 4MU administration time and experimentally treated diseases

Species	Conditions	Treatment duration	Dose [g/kg/day]	PubMed ID	Year
Mouse	Osteoarthritis	8 wks	6 - 10	31774188	2020
	Autoimmune type 1 diabetes	1 wk	10	26368307	2015
	Type 2 diabetes	5 wks	8	34376643	2021
	Obesity and diabetes	22 wks	12	31602424	2019
	Myocardial hypertrophy and fibrosis	8 wks	10	33745300	2021
	Liver fibrosis	16 or 30 days	0.6	31847129	2019
	Pulmonary hypertension	2 wks	0.02	28688167	2017
	Non-alcoholic steatohepatitis	2 wks	0.2	33486695	2021
	Memory enhancement	6 months	6.7	35066096	2022
	Experimental autoimmune encephalomyelitis	5 days	6.7	24973214	2014
	Experimental autoimmune encephalomyelitis	2 wks	6 - 10	27218304	2016
	Renal ischaemia–reperfusion injury	2 or 30 days	10	24078641	2013
	Vascular injury	24 days	6	23484050	2013
	Chronic prostatitis	6 wks	0.5	34320251	2021
	Lung cancer	15 days	0.4 (orally), 0.02 (IP)	35202638	2022
	Malignant peripheral nerve sheath tumor	6 wks	0.4 - 0.5	27706810	2017
	Orthotopic hepatocellular carcinoma	2 wks	1	22038477	2012
	Leukemia	10 days	0.15, 0.3, 0.45	23876826	2013
	Hepatocellular carcinoma	12 months	0.013 - 6.7	29475909	2018
	Hepatocellular carcinoma	12 wks	0.025 - 0.5	30858465	2019
	Hepatocellular carcinoma	20 days	0.4	25882295	2015
	Hepatocellular carcinoma	1 days	0.2	33737571	2021
	Metastatic breast cancer stem–like cells	45 days	0.4	22113945	2012
	Metastatic lesions of bone	14 days	0.0125	21387290	2012
	Bladder cancer	35 days	0.2 - 0.4	28972965	2017
	Pancreatic tumor	4 wks	2	27846148	2017
	Prostate cancer	28 wks	0.45	25868577	2015
	Pancreatic cancer	x	2	27698797	2016
	Acute Lung Injury	2 days	0.45	24141285	2013
Rat	Nerve injury	3 hrs or 7 days	0.4	18725268	2008
	Spinal cord injury	8 wks	2.0	https://doi.org/10.1101/2023.01.23.525137	2023
	Ischemia/reperfusion injury	1 day	0.025	32661417	2020
Human	Covid-19	X	1.2	35304437	2022
